# Luminescent Coatings Based on (3-Aminopropyl)triethoxysilane and Europium Complex β-Diketophosphazene

**DOI:** 10.3390/polym14040728

**Published:** 2022-02-14

**Authors:** Violetta V. Maslennikova, Sergey N. Filatov, Alexey V. Orlov, Nikolay M. Surin, Evgeniya A. Svidchenko, Evgeniy M. Chistyakov

**Affiliations:** 1 D. Mendeleev University of Chemical Technology of Russia, 125047 Moscow, Russia; violetta.1998@mail.ru (V.V.M.); filatovsn@list.ru (S.N.F.); alexeyorlovvladimirovich3829@gmail.com (A.V.O.); 2 Enikolopov Institute of Synthetic Polymeric Materials, Russian Academy of Sciences, 117393 Moscow, Russia; niksurin@yandex.ru (N.M.S.); evgensv@yandex.ru (E.A.S.)

**Keywords:** phosphazene, siloxane, luminescence, thermal resistance, hydrophobicity, adhesion, diketone

## Abstract

The reaction of β-diketophosphazene with the europium (III) salt synthesized the corresponding metal complex which was structured with (3-aminopropyl)triethoxysilane and treated with dibenzoylmethane for additional coordination of europium atoms. The polymer thus obtained exhibits luminescence with a maximum of 615 nm, which is characteristic of europium. The polymer is thermally stable up to 300 °C, the coating based on it has a contact angle of 101°, and the adhesive strength of the coating to non-finished glass (according to ISO 2409: 2013) is 1 point.

## 1. Introduction

Luminescent materials as well as lanthanide complexes have attracted much attention in recent decades due to their wide range of applications in photonics, namely, their use for obtaining tunable lasers, light-emitting diodes, optical amplifiers and luminescent probes for bioanalysis [1,2,3,4,5,6,7,8]. However, the lanthanides show very low absorption coefficients, which requires the use of certain lanthanides for additional excitation of the metal center. This excitation that also known as the “antenna effect” is possessed by coordination ligands which can transfer energy from the ligand to the metal center resulting in luminescence of lanthanide ions [9]. A large number of special ligands have been synthesized for sensitizing the luminescence of metals [10,11,12,13,14,15,16,17], in particular, europium [18,19,20]. Among them β-diketones are excellent chelating ligands used to sensitize the luminescence of the europium ion and have proven themselves well [10,21,22]. For example, a study was presented to research the influence of structural effects on the photophysical properties of mono-β-diketonate and bis-β-diketonate complexes of Eu (III). As a result of this study, it was found that bis-β-diketones still have advantages for the construction of highly luminescent lanthanide complexes compared to mono-β-diketones [23,24]. Nevertheless, low molecular weight complexes have a number of disadvantages: they are not be able to form films, they sublime when heated, they are highly hygroscopic, hydrophilic or completely soluble in water, and their adhesion is not always satisfactory, thus limiting their areas of application. These problems can be solved by introducing complexes into polymer matrices, but in this case, it is difficult to achieve aggregative stability and the system becomes heterogeneous. Therefore, the polymers that are capable of complexation themselves should be used. Such polymers can be obtained on the basis of organophosphazenes. In addition, depending on the nature of the organic radicals, we can obtain polymers with other positive properties such as high optical and dielectric properties, proton conductivity, fire-resistance, thermal resistance, biocompatibility, hydrophobicity, hydrophilicity, bioactivity, etc. [25,26,27,28,29]. In addition, the lightness of chemical modification of phosphazenes makes it possible to obtain on their basis coordination compounds for luminescent materials [30,31,32], including those based on europium [33,34].

At the same time there are no polymer materials that have a significant range of performance properties, such as thermal stability, high glass transition temperature, complex formation, good adhesion to glass, resistance to moisture, and resistance to luminescence, which are important for modern optical electronics. Therefore, the goal of this work is to develop a polymer capable of meeting all of these requirements. The use of aryloxyphosphazene as a base is aimed at achieving high thermal stability. The β-diketogroups in the composition of phosphazene should form a complex with europium capable of luminescence. Structuring of this complex with (3-aminopropyl)triethoxysilane should simultaneously increase the glass transition temperature and ensure adhesion of the resulting polymer to glass. The polymer itself, in this case, should be hydrophobic due to the absence of highly polar fragments.

These parameters are very important for the use of the developed polymer in materials for OLED technologies, the semiconductor industry and organic solar cells. Adhesion to glass is very important because silica shells are used in semiconductor cells. Hydrophobicity helps to reduce the water permeation rate of the product. Exposure to temperature occurs during the manufacture and operation of the device [35]. Due to the luminescent properties of the polymer, it can be used both in light sources and as a detector.

## 2. Materials and Methods

### 2.1. Materials

Europium (III) chloride, anhydrous, powder, 99.99%; dimethylformamide (DMF), anhydrous, 99.8%; sodium, 99.9% trace metals basis; ethanol, anhydrous, ≥99.5; (3-aminopropyl)triethoxysilane (APTES), 99%; N-methyl-2-pyrrolidone, anhydrous, 99.5%; tetrahydrofuran (THF), anhydrous, ≥99.9%; dibenzoylmethane (DBM), 98%. All reagents manufactured by Sigma-Aldrich chemicals, Saint Louis, MO, USA.

### 2.2. Synthesis of Compound ***1***

The synthesis and characterization of compound **1** are presented in [36].

### 2.3. Synthesis of Compound ***2***

Compound **1** (0.5 g, 0.3663 mmol) was dissolved in 10 mL of DMF, then sodium ethylate was added to it (this is prepared by dissolving 0.051 g, 2.1978 mmol of metallic Na in 3 mL of ethanol) and the reaction mixture was stirred for 1 h. Then a solution of europium (III) chloride (0.28155 g, 0.7326 mmol) with 10 mL of DMF was added to the mixture. The reaction mixture was stirred with a magnetic stirrer for 2 h at room temperature. After that, solution **2** was poured into 50 mL of water. The brown precipitate was filtered and washed repeatedly with water. The final product was dried under vacuum for 2 h at 90 °C. Yield: 95%.

### 2.4. Synthesis of Compound ***3***

Compound **2** (0.5 g, 0.3 mmol) was dissolved in 5 mL of N-methyl-2-pyrrolidone with stirring at 100 °C. After that, the solution was cooled to 50 °C, then (3-aminopropyl) triethoxysilane (0.067 g, 0.3 mmol) and 0.05 mL of water were added to it and the mixture was stirred for 1 h. The solution was used to make samples.

### 2.5. Coating Preparation for Testing Adhesion, Hydrophobicity and Luminescence Properties

The solution prepared in Section 2.4 was dropped onto the quartz glass and evenly distributed on the surface with a glass rod. The glass was placed in a vacuum cabinet and the solvent was evaporated at a residual pressure of 0.1 bar and at temperatures of 80 °C for 3 h and 100 °C for 3 h and 150 °C for 6 h.

### 2.6. Preparation of Samples for IR Spectroscopy, TGA and DSC

Samples were prepared in the same way as in Section 2.5, but a Teflon substrate was used instead of quartz glass.

### 2.7. Synthesis of Compound **4**

The film (~0.1 g) was boiled in 100 mL of 0.01 M DBM solution for 20 h.

Elemental composition of compound **4** (wt.%): C 53.65; N 3.70; O 17.25; Si 1.83; P 5.67; H 4.66; Eu 13.24.

### 2.8. Methods

Differential scanning calorimetry (DSC) measurements were performed using the NETZSCH STA 449F1 (Erich NETZSCH GmbH & Co. Holding KG, Selb, Germany) instrument (10 °C min^−1^). Argon was used as a purge gas.

The thermogravimetric analysis (TGA) were conducted on a Derivatograph-C instrument (MOM SZERVIZ KFT., Budapest, Hungary) under argon using the samples of ~10 mg at the heating rate of 10 °C min^−1^.

The IR spectra were recorded on the FTIR spectrometer Nicolet 380 (Thermo Fisher Scientific, Waltham, MA, USA) with the FTIR prefix spectrometer in the transmission mode in the range 4000–400 cm^−1^.

Adhesion was measured in accordance with ISO 2409:2013.

Water contact angles were measured using the Goniometer LK-1 and “Drop Shape” software.

Elemental analysis was performed using a scanning electron microscope JEOL 1610LV (JEOL Ltd., Tokyo, Japan) with energy dispersive spectrometer for electron probe microanalysis SSD X-Max Inca Energy (Oxford Instruments, Abingdon, UK).

Luminescence and luminescence excitation were measured on an ALS-01M spectrofluorometer (ISPM RAS, Moscow, Russia) with intensity suppression of light reflected and scattered by the sample.

Film sample was placed in N-methyl-2-pyrrolidone and stirred slowly for 3 days to determine the gel fraction. After that, the solvent was poured off, the sample was washed with THF with stirring for 2h, the film was separated by decantation, and it was dried in vacuum for 6 h at 150 °C.

## 3. Results and Discussion

**Figure 1:**
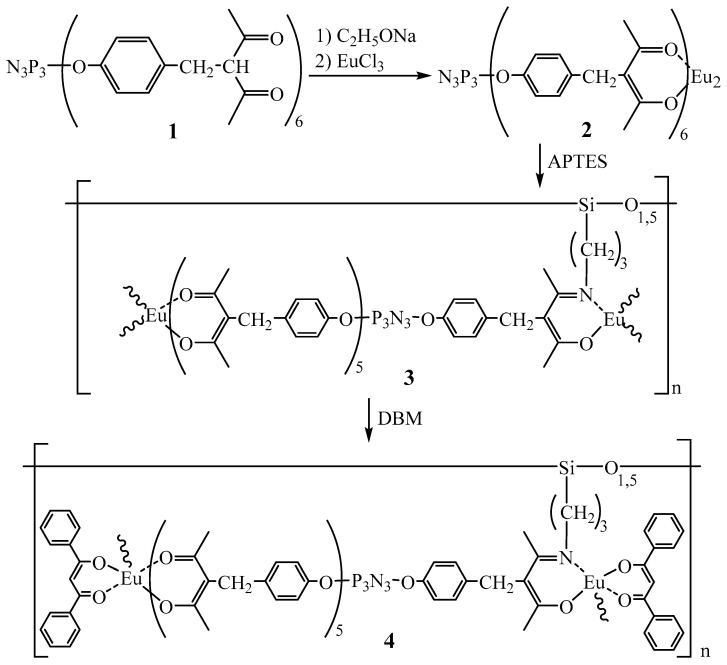
Scheme of the synthesis of compounds **1**–**4**.

The synthesis of the europium-containing polymer (compound **4**) was performed in several steps according to the scheme shown in Figure 1. Firstly, the europium complex (compound **2**) was obtained from compound **1**, which turned out to be soluble only under heating in N-methyl-2-pyrrolidone, which determined the further strategy of synthesis and processing of all obtained products.

**Figure 2:**
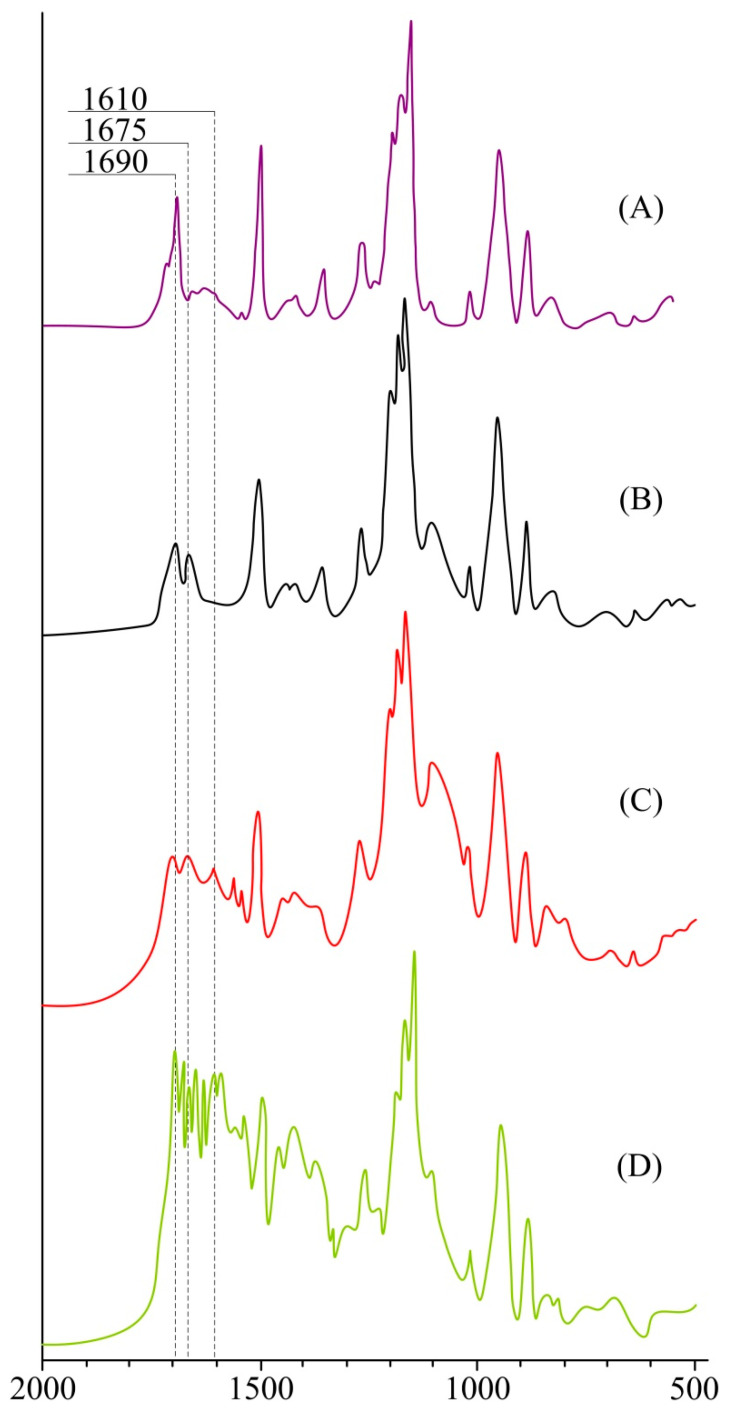
IR spectra compounds **1**–(**A**), **2**–(**B**), **3**–(**C**) and **4**–(**D**).

On IR spectra **2** (Figure 2B) we can notice the presence of valence vibrations of diketone carbonyl groups that are involved in metal coordination in the region of 1675 cm^−1^, which are absent in the spectra of compound **1** (Figure 2A), confirming the formation of a metal complex with europium.

**Figure 3:**
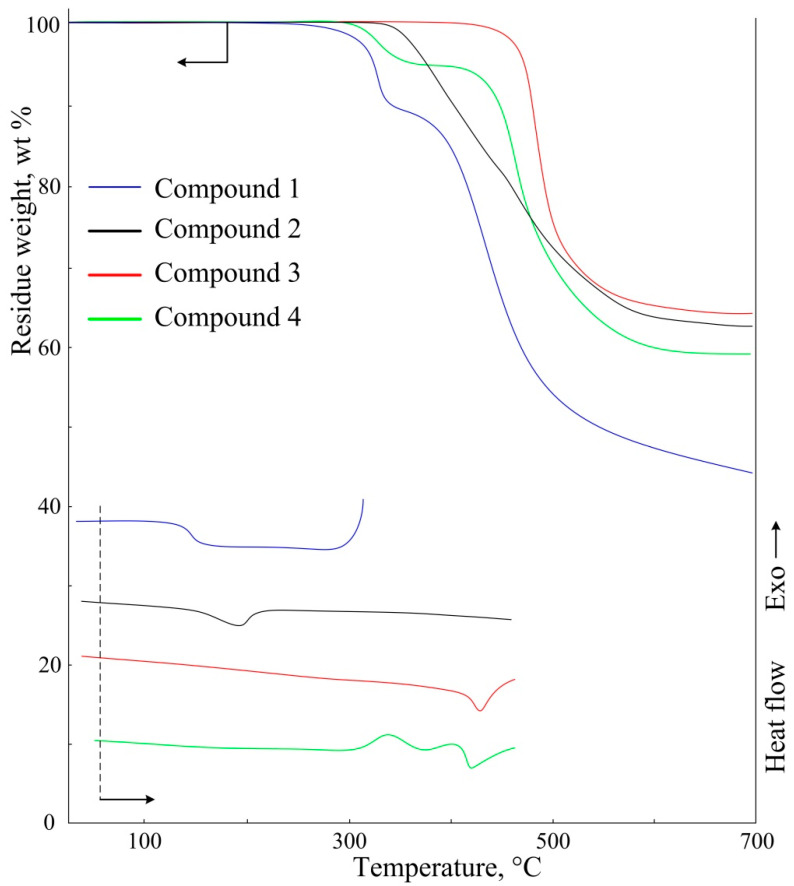
TGA and DSC curves of the obtained compounds.

The temperature at the beginning of the decomposition of compound **2** which was determined by TGA method (Figure 3) is 320 °C. This is 100 °C higher than the start of decomposition of the original **1**, which is caused by the formation of additional bonds by coordinating the metal with β-diketogroups. There is also an increase in the glass transition temperature, which is 40 °C higher than in sample **1** and this amounts to 170 °C. Also, the cox residue of the metal complex at 700 °C is 20% higher than that of the initial ligand.

(3-aminopropyl)triethoxysilane was used to structure the metal complex to form a crosslinked polymer. This compound was chosen due to the possibility of its homocondensation with the formation of silsesquioxane structures and, at the same time, the ability to interact with the diketogroups of the ligand and its metal complexes, thus giving the corresponding azomethines. The formation of azomethine groups is confirmed by the IR spectra of the obtained polymer compound **3** (Figure 2C). The spectrum exhibits a peak at 1610 cm^−1^ related to the valence vibrations of C=N, which is absent from the spectra of compounds **1** and **2**.

There is the increase of thermal characteristics of the obtained polymer **3** due to the structuring of complex **2**. Its glass transition temperature is 400 °C, close to the temperature of the beginning of decomposition. Coke yield at 700 °C reaches 65%.

When studying the luminescence of compounds **2** and **3**, it was found that their quantum yields were extremely low, which is due to the nature of the β-diketogroups of compound **1**, which are fragments of acetylacetone. The methyl groups of acetylacetone have little effect on the energy transfer between the ligand and the metal; it was therefore decided to additionally treat the polymer **3** with dibenzoylmethane. After the polymer treatment the polymer mass increased by 5%, which indirectly indicates its additional coordination with dibenzoylmethane. This conclusion was confirmed by the IR spectra, which show a change in the nature of the valence vibrations of C=O groups in the region 1620–1690 cm^−1^. There are also a number of typical DBM signals: 1590, 1300, 1230 cm^−1^ in the spectra of the obtained compound **4** (Figure 2D).

**Figure 4:**
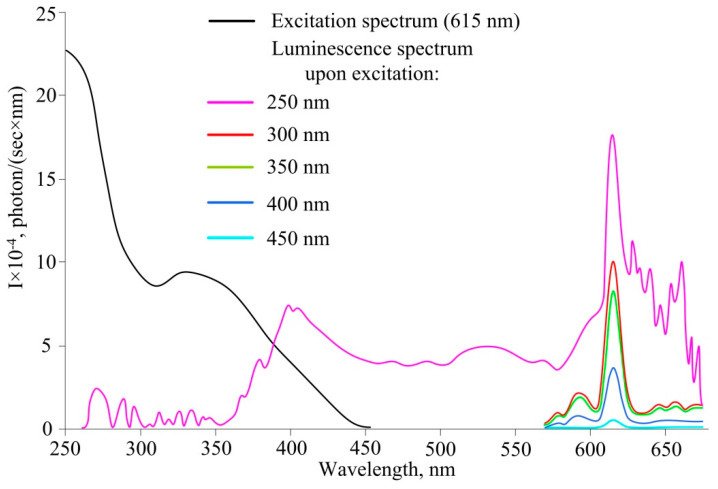
Excitation spectrum (recorded at 615 nm) and luminescence spectrum of compound **4**.

The excitation spectrum and luminescence spectrum of compound **4** are shown in Figure 4.

Upon excitation with light with wavelengths of 300–450 nm, the luminescence spectrum contains the characteristic luminescence of europium (max. 615 nm). Upon excitation by light with a wavelength of more than 450 nm, there is no emission of europium. The measurements showed that there is a transfer of the electronic excitation energy from the ligand to Eu. This is evidenced by the independence of the spectral distribution of emission lines in the range of 575–650 nm.

It can be seen from the luminescence spectrum with excitation at 250 nm that there is broadband luminescence in addition to the lines related to the emission of europium. The band with a maximum at about 400 nm is probably related to the intrinsic fluorescence of the ligand. Since the ligand has a large number of conjugated systems, namely aromatic, phosphazene rings and β-diketogroups, they are capable of luminescence in a wide range of wavelengths under the influence of ultraviolet irradiation.

On the DSC curve of polymer **4** there is an exothermic peak in the region of 300–370 °C. At the same time, on the TGA curve there is a 5% step of mass loss, which is caused by the decomposition of complex **3** with DBM and evaporation of the DBM. Upon further heating, the polymer is stable up to the temperature of 400 °C. At the same time, on the DSC curve there is devitrification of the polymer at this temperature, which corresponds to the temperature characteristics of polymer **3**. A difference of 5 wt.% is also observed in the coke residues of compounds **3** and **4**.

Compound **4** is resistant to solvents. During sequential extraction with N-methyl-2-pyrrolidone and THF the soluble fraction was about 1%. This indicates both the formation of the cross-linked polymer and the stability of the polymer **3** complex with DBM.

**Figure 5:**
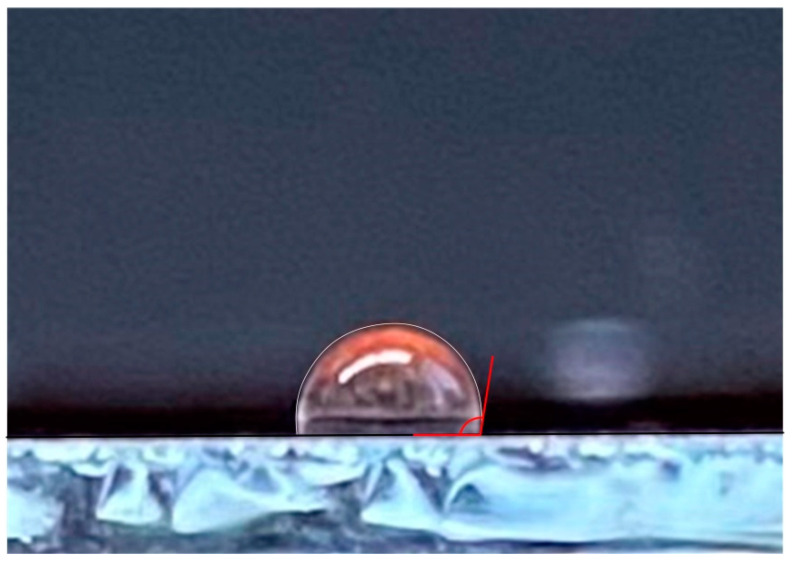
A picture of a drop of water on the surface of the compound **4**.

In a study of the hydrophobicity of the coating based on compound **4** the wetting angle was 101° (Figure 5).

Despite the fact that the polymer is highly hydrophobic, when applied to glass it forms a coating with an adhesive strength of 1 point. Such high adhesion to non-finished glass can be explained by the coordinating action of the Si-OH groups on its surface.

## 4. Conclusions

As a result of the research, a polymer was obtained that can be used in the form of films and coatings as a source of monochromatic red light with a wavelength of 615 nm. In addition, the synthesized polymer can be used in the manufacturing of UV sensors and membranes of analytical electrodes, in laser and space devices and other devices operating under extreme conditions due to its high thermal stability (up to 300 °C), resistance to water and solvents.

## Data Availability

Not applicable.
